# GC/MS-Based Metabolomics Reveals Biomarkers in Asthma Murine Model Modulated by* Opuntia humifusa*

**DOI:** 10.1155/2018/1202860

**Published:** 2018-11-01

**Authors:** Seung-Ho Seo, Eun-Ju Kim, Seong-Eun Park, Sung-Hoon Byun, Soon-Young Lee, So-Hyeon Bok, Dae-Hun Park, Hong-Seok Son

**Affiliations:** ^1^School of Korean Medicine, Dongshin University, Naju 58245, Republic of Korea; ^2^Department of Oral and Maxillofacial Surgery, Gyeongsang National University Hospital, Jinju 52727, Republic of Korea

## Abstract

GC/MS coupled with multivariate statistical analysis was performed to identify marker metabolites in serum of mice after healing ovalbumin- (OVA-) induced asthma using* Opuntia humifusa*. Principal component analysis (PCA) score plot showed separation among groups, with metabolite profiles of serum showing differences according to various treatments for the asthma murine model. Levels of stearic acid and arachidic acid were significantly lower in the serum from OVA-induced group than those from the control group. Dexamethasone treatment group was characterized by higher serum levels of urea, myristic acid, and palmitic acid along with lower levels of aspartic acid compared to OVA-induced group.* O. humifusa* treatment mice groups showed dose-proportional higher levels of urea and glycerol than OVA-induced group. These results highlight that GC/MS-based metabolomics is a powerful technique for identifying molecular markers of asthma.

## 1. Introduction 

Asthma is one of the most common chronic inflammatory disease of the airways [1, 2]. Despite the fact that the majority of asthma cases can now be well controlled by current medications, asthma still accounts for over 250,000 deaths annually worldwide [3]. Current treatments for asthma are mainly performed by suppressing airway inflammation with inhaled corticosteroids and relieving bronchoconstriction with bronchodilators [4]. Although this treatment improves symptoms of asthma, prolonged use of inhaled corticosteroids has been associated with side effects, including osteoporosis [5–8], hypothalamic‐pituitary‐adrenal axis suppression [9], immune suppression [10], mood changes [11], and pharyngitis [12]. These issues underscore the need to develop new antiasthmatic drugs with few or no adverse effects.

Treatment of asthma by natural products such as herbal medicine can increase treatment effects with reduced side effects. Natural products such as grape seed extract [13],* Garcinia* [14],* Curcuma longa* [15], and* Rosmarinus officinalis* [16, 17] have shown efficacy in treating asthma without side effects. Among them,* Opuntia humifusa* extracts can potently modulate expression levels of inflammatory cytokines [18, 19]. In our previous study, we have found that* O. humifusa* possesses dose-dependent antiasthmatic effects [20]. However, it is difficult to determine functional substances in* O. humifusa* and mechanisms involved in its pharmacological effects. The role of molecular determinants as mediators of asthma has not been fully understood yet [21].

Metabolomics is based on systematic analysis of a panel of small metabolites, including sugars, amino acids, organic acids, and lipids. Metabolomics is well-suited for studying diseases because metabolite changes represent an integrated pathophysiologic profile [22]. Metabolomics studies have been performed to diagnose diseases with unknown mechanisms such as inflammatory bowel disease [23, 24] and rheumatoid arthritis [25–27]. Many metabolomics studies have also been conducted to derive mechanisms for treating diseases such as cancer [28], autoimmune disease [29], and chronic disease [30, 31]. For example, gas chromatography (GC) mass spectrometry- (MS-) based metabolomics studies have been performed to discriminate subjects with asthma from healthy populations and uncover some potential disease-relevant serum biomarkers [32, 33]. Therefore, metabolic profiles can provide insights into biologic mechanisms of asthma. In our previous study, we have confirmed that* O. humifusa* can dose-dependently suppress morphological changes typically observed in asthma [20]. In this study, a GC/MS-based metabolomic approach was employed in a profiling mode to reveal differences in key metabolites of serum of mice after healing ovalbumin-induced asthma using* O. humifusa*.

## 2. Materials and Methods

### 2.1. *O. humifusa* Collection and Extraction

*O. humifusa* leaves were obtained from Wando Arboretum in Jeonnam province, Korea. Ten grams of dried leaves were extracted twice with 100 mL of 80% ethanol at room temperature. The extract solvent was evaporated, dried, and stored at -50°C until use.

### 2.2. Experimental Animals and Serum Collection

Animal experiment and serum collection were conducted as described previously [20]. Eighty-four 6-week-old female BALB/c mice were purchased from Samtako (Osan, Korea). These mice were kept in standard laboratory conditions with a room temperature of 24 ± 1°C, humidity of 65 ± 5%, and a controlled light/dark cycle (12/12 h). After 6 days, these mice were divided into six experimental groups according to treatment: (1) vehicle control (sterilized tap water), (2) ovalbumin- (OVA-) induced asthma model, (3) 1 mg/kg/day dexamethasone with OVA induction, (4) 50 mg/kg/day* O. humifusa* with OVA induction, (5) 100 mg/kg/day* O. humifusa* with OVA induction, and (6) 500 mg/kg/day* O. humifusa* with OVA induction. All mice were sensitized by intraperitoneal injection with 20 *µ*g OVA and 1 mg aluminum hydroxide hydrate (Sigma-Aldrich) in 500 *μ*L saline on days 1 and 8. Asthma induction in mice was performed by inhaling 5% OVA for 30 min once daily using a nebulizer (3 mL/min, NE-U17, Omron, Kyoto, Japan) for 5 consecutive days (21-25 days). During the same 5-day period, the vehicle control group was exposed to saline and aluminum hydroxide hydrate with a nebulizer. Mice in treatment groups were treated once daily with oral doses of 1 mg/kg/day of dexamethasone, 50 mg/kg/day of* O. humifusa*, 100 mg/kg/day of* O. humifusa, *and 500 mg/kg/day of* O. humifusa *in sterilized tap water 1 h prior to OVA challenge. On the 6th day, all animals were induced anesthesia using 50 mg/kg Zoletil (Virbac, Carros, France). To obtain serum, whole blood was collected and centrifuged at 12,000 rpm for 15 min at 4°C (Sorvall Legend Micro 17R, Thermo Fisher Scientific, Waltham, MA, USA).

All experiments were approved by the Institutional Animal Care and Use Committee (IACUC) of Chonnam National University (Animal Study Approval No. 201 CNU IACUC-YB-R-2015-50).

### 2.3. Sample Preparation and GC-MS Derivatization

Serum (100 *µ*L) was thawed at 4°C and mixed with methanol (300 *µ*L) to precipitate proteins. The mixture was vigorously vortexed for 15 s. After centrifuging samples at 13,000 rpm for 5 min, 300 *µ*L of the supernatant was lyophilized. One hundred *µ*L of O-methoxamine hydrochloride in pyridine (15 mg/mL) was then added into the freeze-dried sample. After vortex-mixing each sample for 3 min at room temperature, 100 *µ*L of N,O-bis-(trimethylsilyl)-trifluoroacetamide (BSTFA) containing 1% trimethylchlorosilane (TMCS) was added and incubated at 70°C in a water bath for 1 h. Finally, 600 *µ*L of methyl stearate (10 mg/L in heptane) was added as an internal standard. After centrifuging at 13,000 rpm for 5 min, supernatant was transferred to a 1.5 mL glass vial for GC/MS analysis.

### 2.4. GC-MS Analysis Condition

Chromatography analysis was performed with a QP-2020 Gas Chromatography Mass Spectrometer (Shimadzu, Kyoto, Japan). Separation was achieved using a Rtx-5MS capillary column (0.25 mm × 30 mm × 0.25 *μ*m) in a split mode (1:30). The initial GC oven temperature was kept at 60°C for 1 min. It was increased to 300°C at a heat rate of 10°C/min. It was then held at 300°C for 10 min. Helium was used as the carrier gas and flow rate was kept constant at 1.0 mL/min. MS conditions were as follows: transfer detector line, 280°C; filament ion source, 230°C; and quadrupole temperature, 150°C. Ionization was achieved with an electron beam at 70 eV. MS data were obtained in full-scan mode at a range of m/z 50-550.

### 2.5. Data Processing and Statistical Analysis

GC-MS metabolite data were exported to comma delimited format (CDF) as raw data which were then imported into XCMS website (https://xcmsonline.scripps.edu) for retention time correction, baseline filtration, and alignment. Excel files obtained from XCMS were then imported into SIMCA-P version 14.0 software (Umetrics, Umea, Sweden) for multivariate statistical analysis such as principal component analysis (PCA) and partial least-squares discriminant analysis (PLS-DA). Distinction variables were selected by variable importance in projection (VIP) > 1.2 and* p* < 0.05. When identifying potential biomarkers, retention index (RI) was calculated in proportion to n-alkane (C8-C40) standards. The intensity of identified metabolites was subjected to statistical comparison. T-test was performed to compare the relative amount (peak intensity) of identified metabolites between the two groups. Analyses of variance (one-way ANOVA) followed by Tukey's post hoc multiple comparison tests were also performed for examination of group differences. All statistical analyses were performed using SPSS version 22.0 software package (SPSS Inc., Chicago, IL, USA).

## 3. Results and Discussion

### 3.1. Metabolic Profiling

**Figure 1:**
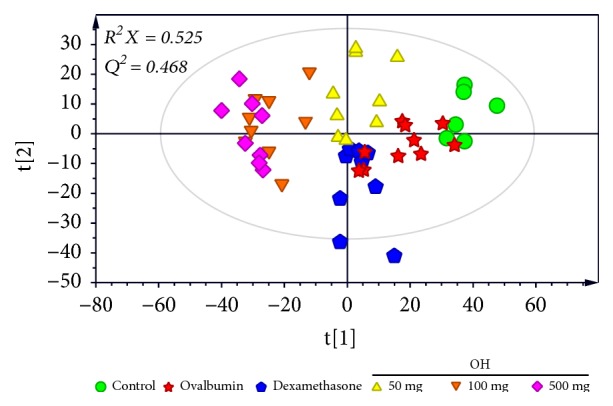
PCA score plot derived from GC/MS data of serum in mice, showing different metabolic profiles according to various treatments in asthma murine model. Symbols with different shapes denote serum samples from different treatments. OH,* Opuntia humifusa*.

After GC/MS data processing using XCMS, a total of 1,407 signal features were obtained. PCA was performed using features obtained from GC-MS data to investigate metabolic differences between samples (Figure 1). When two components were calculated, cumulative* R*^2^*X* and* Q*^2^ values were 0.525 and 0.468, respectively. Although some samples were overlapped in the PCA score plot, samples showed separation pattern between groups, indicating that metabolite profiles of serum samples were different according to various treatments for the asthma murine model. A clear separation of samples between the control group and dexamethasone treatment group in the PCA score plot suggested that metabolic profiles of serum were changed even though symptoms of asthma were recovered by dexamethasone treatment. Interestingly, serum samples of* O. humifusa* treatment groups were clearly distinguished from those of other groups, suggesting that metabolites of serum were changed after* O. humifusa* treatment. Such difference in metabolites might be due to the substances in* O. humifusa* or the metabolites involved in the therapeutic mechanism of asthma.

### 3.2. Different Metabolites Induced by OVA

**Figure 2:**
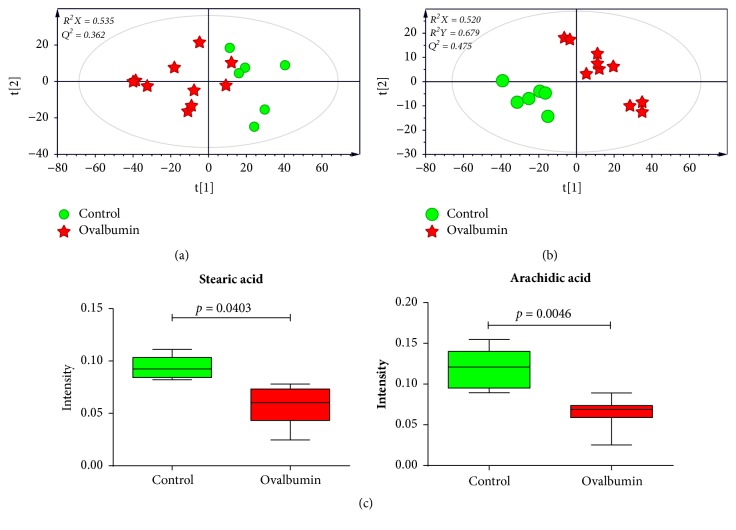
PCA (a) and PLS-DA (b) score plots derived from GC/MS data of serum samples between control and OVA-induced groups, suggesting metabolic changes by OVA induction. Panel (c) shows box plots of identified metabolites contributing to differentiation in the PLS-DA model (VIP > 1.2 and* p* < 0.05). The PLS-DA model was validated by a permutation test (n = 200).

To better visualize subtle similarities and differences after induction of asthma, PCA was performed using features from control and OVA-induced groups. However, samples were not fully distinguishable in the PCA score plot between these two groups (Figure 2(a)). To maximize separation, PLS-DA, a supervised pattern recognition method, was applied for the same datasets. PLS-DA score plot of serum samples showed a clear separation of control samples from OVA-induced asthma samples (Figure 2(b)), with modeling fit* R*^2^*X* values of 0.520 and* R*^2^*Y* values of 0.679. It also showed good prediction* Q*^2^ value of 0.475.

Through a permutation test,* Q*^2^ and* R*^2^ values were found to be higher than their original values, proving suitability and validity of this model. These results suggested that metabolic changes indeed occurred in the serum of asthmatic mice. To identify which metabolites were responsible for the separation of these two groups, variable importance in projection (VIP) score was determined. Based on a VIP score > 1.2 from PLS-DA with* p* < 0.05 in two-tailed Student's* t*-test, stearic acid and arachidic acid were identified as metabolites contributing to the difference. Metabolites were then identified based on fragmentation patterns of GC/MS library, RI value, and other researchers' experimental data using the same GC/MS analysis method [34]. Levels of stearic acid (*p* = 0.0403) and arachidic acid (*p* = 0.0046) were found to be significantly lower in serum from asthmatic mice than that from the control mice (Figure 2(c)).

### 3.3. Different Metabolites by Dexamethasone Treatment

**Figure 3:**
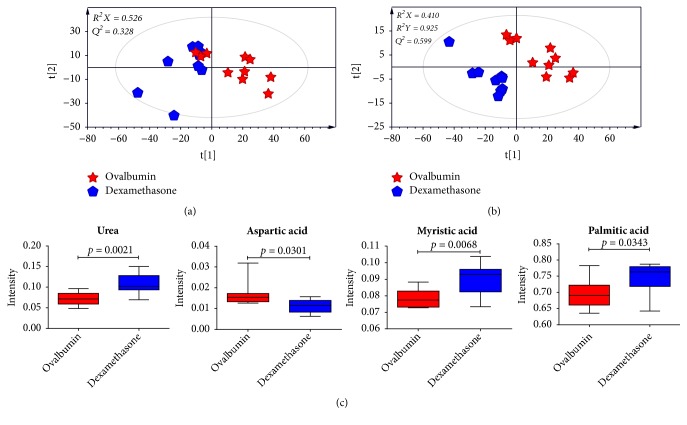
PCA (a) and PLS-DA (b) score plots derived from GC/MS data of serum samples between OVA-induced group and dexamethasone treatment group, suggesting metabolic differences induced by dexamethasone treatment. Panel (c) shows box plots of identified metabolites contributing to differentiation in the PLS-DA model (VIP > 1.2 and* p* < 0.05). The PLS-DA model was validated by a permutation test (n = 200).

To investigate metabolic differences in serum between OVA-induced group and the dexamethasone treatment group, PCA and PLS-DA models were generated (Figures 3(a) and 3(b)). PLS-DA score plot showed a clear separation between OVA-induced mice and dexamethasone treatment mice (*R*^2^*X *= 0.410,* R*^2^*Y *= 0.925, and* Q*^2^= 0.599). The PLS-DA model was validated by a permutation test repeated 200 times. After data filtration based on high VIP score with* p *< 0.05, a total of four metabolites were identified as factors contributing to the discriminating PLS-DA model. The dexamethasone treatment group was characterized by higher serum levels of urea, myristic acid, and palmitic acid along with lower levels of aspartic acid compared to those in the OVA-induced group (Figure 3(c)).

### 3.4. Different Metabolites by* O. humifusa* Treatment

**Figure 4:**
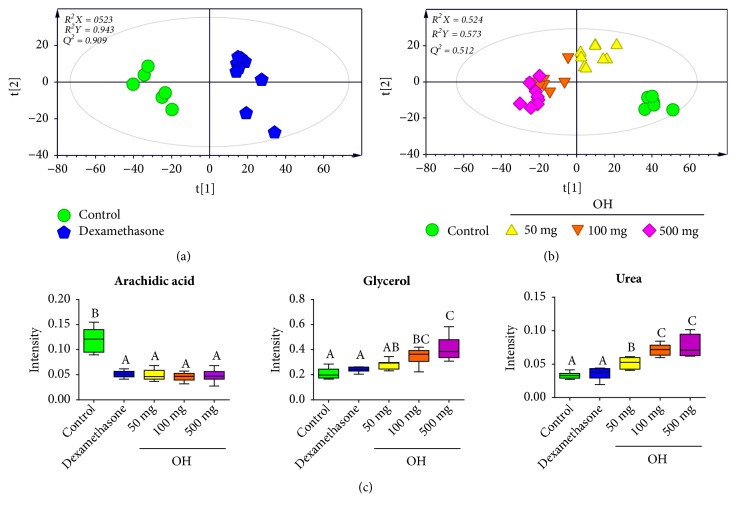
PCA score plots derived from GC/MS data of serum samples between control and dexamethasone treatment group (a) and control and* O. humifusa* treatment group (b), suggesting metabolic differences according to different treatments after OVA induction. Panel (c) shows box plots of identified metabolites contributing to differentiation in the PLS-DA model (VIP > 1.2 and* p* < 0.05). The PLS-DA model was validated by a permutation test (n = 200). OH,* Opuntia humifusa*.

To investigate changes in serum between control and the treatment groups (dexamethasone and* O. Humifusa*), PCA was applied. Unsupervised PCA basically demonstrated a clear separation between the control and the treatment groups (dexamethasone and* O. Humifusa*) (data not shown). To obtain improved segregation and gain better understanding of variables responsible for the fractionation, a supervised clustering PLS-DA model was generated (Figures 4(a) and 4(b)). The score plot of PLS-DA showed a distinct separation, indicating that metabolite profiles in serum of mice were significantly altered by dexamethasone and* O. humifusa* treatment. The treatment groups were characterized by lower serum levels of arachidic acid compared to those in control group. Mice from* O. humifusa* treatment group was found to have dose-proportional higher serum levels of glycerol and urea than mice from control group (Figure 4(c)).

Lipid mediators are key drivers of inflammatory responses in asthma. They have well-characterized roles in T-cell recruitment and energy metabolism [22]. Generally, polyunsaturated fatty acids are implicated in the prevention of various human diseases, including inflammation-associated diseases [35]. However, the effect of saturated fatty acids on inflammatory mechanism is currently unclear. In this study, levels of stearic acid and arachidic acid were decreased by OVA induction while levels of myristic acid and palmitic acid were increased by dexamethasone treatment (Figures 3(c) and 4(c)). Dexamethasone is commonly used as an asthma treatment drug. It can suppress WBCs increases induced by OVA. This effect might be related to inhibition of NF-*κ*B activity by saturated fatty acids. According to Pan et al. [36], stearic acid supplementation has beneficial effect against cholestatic liver injury by decreasing inflammatory cell accumulation as well as suppressing NF-*κ*B activity. Increasing evidence indicates that stearic acid possesses antioxidant and anti-inflammatory potential [37–40]. In contrast, Schaeffler et al. [41] have reported that stearic acid and palmitic acid can induce activity of NF-*κ*B. Thus, additional research is needed to clarify the relationship between mechanisms of asthma and saturated fatty acids.

Urea level in serum is generally increased when asthma develops [42, 43]. In the present study, administration of dexamethasone and* O. humifusa *increased urea levels in serum. These results are similar to a study of Ho et al. [42], showing that serum urea level is decreased with administration of artesunate but increased after administration of dexamethasone. Dexamethasone and artesunate are typical treatment drugs for asthma. Asthma treatment effect of both drugs is related to regulation of arginine metabolism such as conversion of arginine to urea and ornithine [44, 45].

Metabolomics is a good tool to identify biomarkers of asthma and improve our understanding of the pathophysiology of asthma. Some metabolites (mainly organic acids and amino acids) associated with asthma onset or severity have been identified by many studies [22]. However, these metabolites were not replicated between asthma studies. In the present study, some saturated fatty acids were identified as main metabolites associated with asthma. However, they were rarely noted metabolites in other asthma studies. These differences might be due to differences between animal and clinical trials that can affect the metabolome. Further researches are needed to identify how some metabolites correlate with the mechanism of outbreak and treatment of asthma.

## 4. Conclusions

This study shows that GC/MS-based metabolomics is a powerful technique to determine biomarker metabolites of asthma. In this study, metabolic profiles of serum in mice were different among groups, indicating that metabolites of serum might change according to asthma induction and different treatments. Although some metabolites were identified as metabolites associated with asthma induction and treatment, it is difficult to determine mechanisms involved in change of metabolites. Further metabolomic studies including more samples are needed to understand biologic mechanisms of asthma.

## Data Availability

The data used to support the findings of this study are available from the corresponding author upon request.
